# A Study on the Robustness and Stability of Explainable Deep Learning in an Imbalanced Setting: The Exploration of the Conformational Space of G Protein-Coupled Receptors

**DOI:** 10.3390/ijms25126572

**Published:** 2024-06-14

**Authors:** Mario A. Gutiérrez-Mondragón, Alfredo Vellido, Caroline König

**Affiliations:** 1 Computer Science Department, Intelligent Data Science and Artificial Intelligence (IDEAI-UPC) Research Center, Universitat Politècnica de Catalunya, 08034 Barcelona, Spain; mario.alberto.gutierrez@upc.edu (M.A.G.-M.); avellido@cs.upc.edu (A.V.); 2 Centro de Investigacion Biomédica en Red (CIBER), 28029 Madrid, Spain

**Keywords:** imbalanced data, explainable artificial intelligence, imbalance classification metrics, deep learning, G protein-coupled receptors, molecular dynamics

## Abstract

G-protein coupled receptors (GPCRs) are transmembrane proteins that transmit signals from the extracellular environment to the inside of the cells. Their ability to adopt various conformational states, which influence their function, makes them crucial in pharmacoproteomic studies. While many drugs target specific GPCR states to exert their effects—thereby regulating the protein’s activity—unraveling the activation pathway remains challenging due to the multitude of intermediate transformations occurring throughout this process, and intrinsically influencing the dynamics of the receptors. In this context, computational modeling, particularly molecular dynamics (MD) simulations, may offer valuable insights into the dynamics and energetics of GPCR transformations, especially when combined with machine learning (ML) methods and techniques for achieving model interpretability for knowledge generation. The current study builds upon previous work in which the layer relevance propagation (LRP) technique was employed to interpret the predictions in a multi-class classification problem concerning the conformational states of the β2-*adrenergic* ($\beta_{2}$AR) receptor from MD simulations. Here, we address the challenges posed by class imbalance and extend previous analyses by evaluating the robustness and stability of deep learning (DL)-based predictions under different imbalance mitigation techniques. By meticulously evaluating explainability and imbalance strategies, we aim to produce reliable and robust insights.

## 1. Introduction

G protein-coupled receptors (GPCRs) have, for decades, been the target of intensive research due to their ability to interact with a broad range of chemical partners—from hormones to neurotransmitters [1,2]. Protein receptors such as GPCRs are composed of amino acids whose sequence and arrangement dictate their structural and functional properties. Among these, adrenergic receptors (ARs) offer crucial insights into receptor activation and signaling pathways. Signaling refers to a sequence of structural changes that facilitate the transduction of signals from outside the cell to the cell’s interior [3,4,5]. This process is critical for allowing cells to perceive and appropriately respond to their environment, spanning a broad range of processes, from adaptive responses to disease associations [6,7].

Beta-adrenergic receptors (β-ARs)—a class of GPCR—are of particular significance. They are widely expressed in human cells and extensively studied due to their critical roles in mediating responses to epinephrine and norepinephrine [8]. These neurotransmitters also function as hormones, activating β-ARs to regulate a variety of physiological responses. This interaction has been pivotal in developing targeted therapies for managing conditions such as hypertension, heart failure, arrhythmias, and asthma, thereby profoundly impacting our ability to treat complex diseases [9]. To date, three subtypes of β-AR have been identified in various tissues: β1-, β2-, and β3-ARs. Each subtype exhibits unique effects influenced by their specific tissue distribution and the particular signaling pathways they activate [10,11,12]. For example, β1-ARs are primarily involved in cardiac function, influencing heart rate and contractility, while β3-ARs play a role in fat metabolism. This study, however, focuses on the β2 receptor, which is primarily located in smooth muscle tissues such as the lungs, playing a crucial role in breathing and managing airflow. Additionally, β2-ARs are found in the vascular system and other tissues like the liver and skeletal muscle, where they regulate blood flow and facilitate metabolism. Given their strategic distribution across vital systems, β2 receptors are particularly significant as targets for drugs used in treating respiratory diseases, cardiovascular conditions, and other related disorders [13,14].

A defining characteristic of all GPCR families is their alpha-helical structure, which consists of seven transmembrane helices (7TM) that traverse the cell membrane [2]. These helices are interconnected by intracellular and extracellular loops, with the N-terminus oriented toward the extracellular space and the C-terminus located in the cytosol, defining the beginning and end of the structure. In addition of their specific configuration, all GPCRs share a common signaling mechanism. Upon ligand binding—such as hormones, neurotransmitters, or sensory stimuli—the GPCR undergoes a conformational change that facilitates interaction with the G protein complex, composed of three protein subunits: Gα, Gβ, and Gγ. This interaction triggers the exchange of GDP (guanosine diphosphate) for GTP (guanosine triphosphate) on the Gα subunit, thereby activating it. GDP is a low-energy molecule that, when replaced by the high-energy GTP, activates the Gα subunit. Once activated, Gα-GTP dissociates from the Gβ and Gγ subunits, which then initiate distinct signaling pathways [15,16,17,18].

GPCRs are highly dynamic entities that undergo intricate conformational changes, even in the absence of external stimuli [19]. These receptors transit through multiple conformational states, impacting their activation, downstream signaling, and ultimately, the cellular responses they control [20,21,22]. The recognition of structural motifs, which are recognizable and frequently conserved patterns within these receptors, is key to understanding these conformational transitions. Identifying the residues that constitute relevant motifs can offer insights into the receptor’s function and its potential interactions with other molecules, significantly impacting our understanding of human health and disease. Deciphering these nuances is crucial knowledge for developing more precise and effective therapeutic drugs [17,23]. As such, the vast conformational landscape that GPCRs traverse during their activation pathway remains a primary focus of current research, highlighting the dynamic nature of these receptors.

Our understanding of the conformational dynamics of GPCRs and their influence on transition pathways has been historically limited by gaps in experimental and computational knowledge [24]. However, this gap has significantly narrowed thanks to advances in experimental techniques that have enhanced our ability to capture the intricate dynamics of GPCR activation and regulation [25,26]. Initially, X-ray crystallography was pivotal in shaping our understanding of the structural details of GPCRs, offering precise snapshots of receptor conformations at high resolution that were invaluable for studying complex processes such as the ligand-mediated signaling pathways [27]. Subsequently, Cryo-Electron Microscopy (cryo-EM) enabled the visualization of GPCRs in environments closer to their natural physiological state, thus boosting our ability to capture a broader array of conformational states [28]. This technique has proven particularly valuable for identifying stable conformations previously inaccessible with X-ray crystallography, thereby enhancing our understanding of the structural landscape of GPCRs. Despite these advances, the dynamic nature of GPCR activation, involving rapid and transient conformational changes, remained elusive until the application of molecular dynamics (MD) simulations. These simulations have been pivotal in providing a dynamic view of the motions of atoms and molecules in real time, offering deep insights into the stability, flexibility, and functional dynamics of GPCRs across timescales from milliseconds to seconds [29]. This detailed information has been crucial for understanding the complex landscape of activation pathways, from ligand binding to G-protein coupling [30,31]. MD simulations effectively fill the gaps left by static imaging techniques, yielding insights that were previously unattainable [16,32].

Nevertheless, computational simulations inherently pose challenges due to the vast amounts of data they generate—ranging from terabytes to petabytes [33]. The task of effectively analyzing this wealth of information to generate valuable insights into the mechanisms of complex and dynamic systems such as GPCRs is particularly challenging. In this context, the synergy of MD with ML becomes crucial [34,35,36]. By integrating the high-resolution temporal and spatial data from MD simulations with the advanced pattern recognition and predictive capabilities of ML, researchers can not only process and analyze large datasets more effectively, but also uncover and predict complex molecular interactions and their biological implications with unprecedented accuracy. For instance, supervised deep learning models have been proposed for classifying ligand-determined GPCR conformational properties [37], and further insights about protein–ligand complexes from MD simulation trajectories have also been revealed in [38,39,40]. The applications of ML and deep learning methods to the analysis of MD simulations are diverse and continually expanding [41,42].

In our previous work, we leveraged MD simulations data originally generated on the Google Exacycle platform, as reported by Kohlhoff et al. [43]. Our aim was to uncover relevant motifs associated with specific conformational—active, inactive, and intermediate—as these states are critical to understanding receptor functionality in pharmacological contexts [44]. The pharmacological relevance of these states lies in their potential to reveal interaction points for drug binding, influencing receptor behavior and therapeutic outcomes [2,45]. Active states are typically associated with agonist binding that promotes favorable cellular responses, making them prime targets for drugs aiming to enhance receptor activity [46]. In contrast, inactive states often bind antagonists that inhibit receptor signaling, useful for conditions where receptor suppression is desired. The intermediate states are particularly interesting as they may represent a balance between activation and inhibition, offering nuanced control over receptor signaling pathways [47].

We addressed a supervised problem using deep learning-based models in which explanations from the model predictions becomes critical; it not only validates predictions but also deepens our understanding of structural motifs—related to particular residues—that drive conformational changes in the receptor. Particularly, the concept of Explainable Artificial Intelligence (XAI) has gained increased attention in recent years due to its potential to demystify model reasoning, build trust, enhance transparency, and make AI systems more reliable and understandable [48,49,50]. Additionally, providing explanations for AI decisions can reveal new insights and patterns that were previously hidden within complex data. In domains like biology and pharmacology, this can lead to discoveries and innovations that were previously unreachable and that traditional methods may miss [51,52,53,54].

In this work, we focus on two primary goals. First, we explore the robustness and stability of different explainability techniques for enhancing trustworthiness, transparency, and generalizability of the insights derived from AI models, thereby supporting the broader acceptance of these type of models in scenarios wherein these insights can have significant impacts. In the same line, we also suggest an *intersection of XAI methods* approach, combining insights from multiple explanation methods to derive more reliable or robust interpretations of a model’s behavior. When multiple XAI techniques concur on the significance of the same set of features or patterns, the confidence in the identified features is enhanced, and the associated uncertainty is reduced [55,56,57].

Second, we address the significant challenge of effectively managing the highly imbalanced distribution of our dataset. Effectively handling data imbalance is crucial as it ensures that the model performs well across all classes, preventing biases toward more prevalent classes and enabling the accurate identification of critical features within the receptor. In this context, the correct evaluation of the model performance across imbalance mitigation methods is critical as it helps to determine the most effective techniques for improving model generalization and reliability, ensuring that underrepresented classes are accurately predicted and the derived insights are trustworthy.

## 2. Results

**Figure 1:**
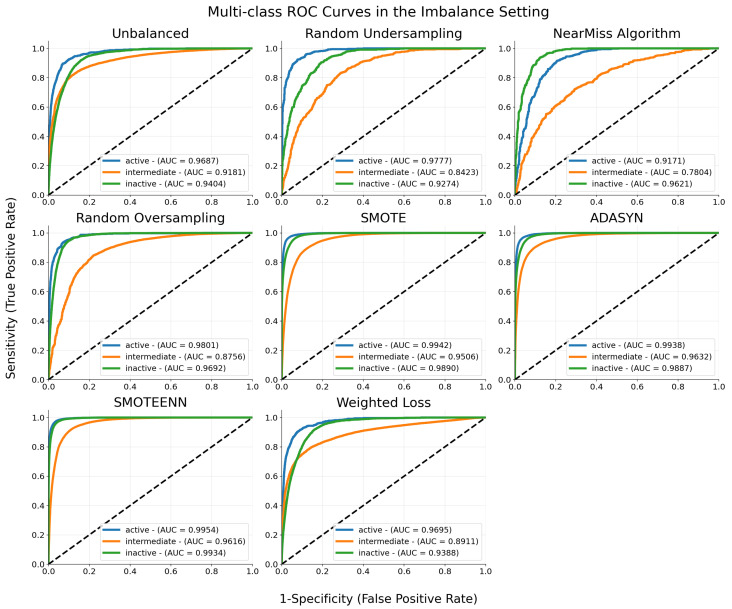
Multi-class ROC curves for each of the analytical settings evaluated. The Area Under the ROC Curve (AUC) values summarize the performance of the models for the three conformational states. A score of 1 indicates perfect classification performance. The black dashed line indicates random classification (AUC = 0.5).

Figure 1 presents the receiver operating characteristic (ROC) curves for our proposed one-dimensional convolution neural network (1D-CNN). The figure illustrates the model’s performance across various class-imbalance mitigation techniques for the three conformational states—active, inactive, and intermediate. This compilation allows for a comparative analysis of how each mitigation strategy affects the model’s ability to distinguish between different states, highlighting the effectiveness of each approach in handling data imbalance.

**Figure 2:**
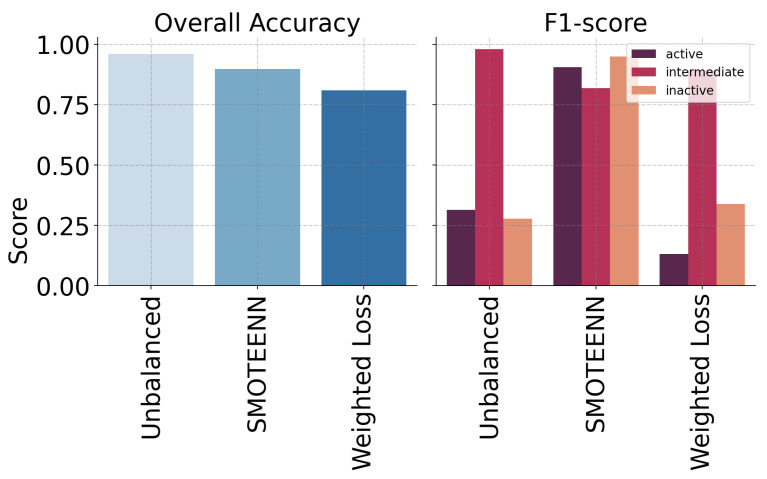
Accuracy and F1-score are displayed for each SMOTEENN and Weighted Loss methods versus an unbalanced distribution of the data. The gross training accuracy is displayed in the left plot, while F1-score is displayed per class in the other plot.

Figure 2, in turn, provides a comparison of the performance of two selected methods, namely SMOTEEN and Weighted Loss, against the evaluation of the model under an unbalanced distribution using the accuracy and F1-score metrics.

**Figure 3:**
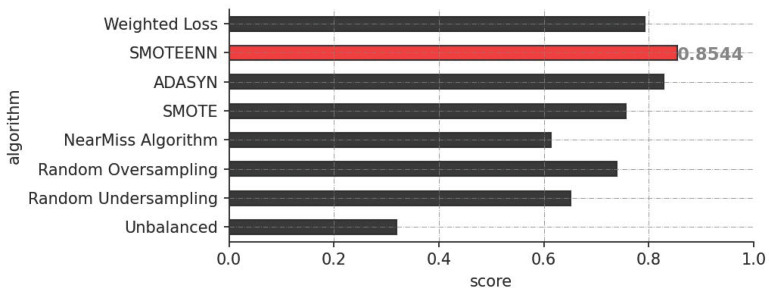
Classification performance as assessed by MCC across various methods for mitigating class imbalance.

To enhance our assessment of the model’s performance, the Matthews Correlation Coefficient (MCC) score provides a more balanced measure compared to ROC curves, particularly in the context of imbalanced datasets. Figure 3 underscores the effectiveness of the class imbalance mitigation methods, demonstrating how they enhance the predictive power of our model and reinforce the significance of these techniques in our analysis.

In addition, we compare our 1D-CNN with simpler models, namely Decision Trees (DT) and Random Forest (RF). For this comparative analysis, we employ precision, recall, and F1-score metrics, which provide a comprehensive evaluation across models. The results are summarized in Table 1. For this comparison, only outcomes from models trained using the SMOTEENN imbalance mitigation technique were included, given its superior performance as shown in Figure 1 and Figure 3. This focused approach ensures a clear and concise understanding of the models’ performance, highlighting the robustness of our 1D-CNN model compared to DT and RF under the best-performing imbalance mitigation strategy.

**Figure 4:**
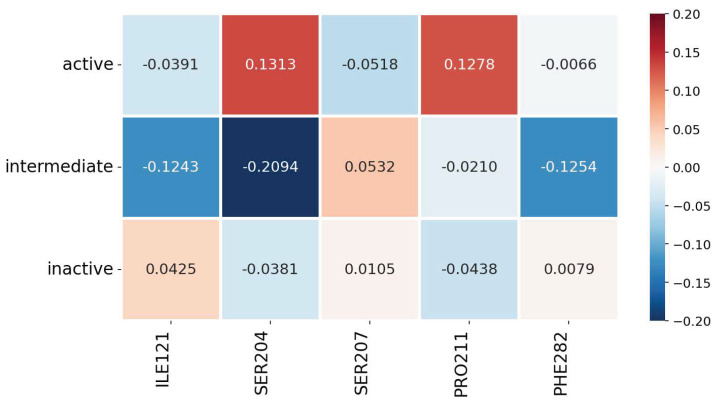
Quantification of the contributions of critical residues, as identified in the literature and analyzed using Layer-wise Relevance Propagation (LRP). Positive contributions to a conformational state are depicted in red, while negative contributions are depicted in blue.

In the context of our explainability study, Figure 4 shows the contributions of some key residues across all imbalance mitigation methods, as analyzed using the Layer-wise Relevance Propagation (LRP) explanation technique. These residues are well-documented in the literature for their crucial roles in the transition of the $\beta_{2}$AR from its inactive to its active state upon binding with a full agonist [58].

**Figure 5:**
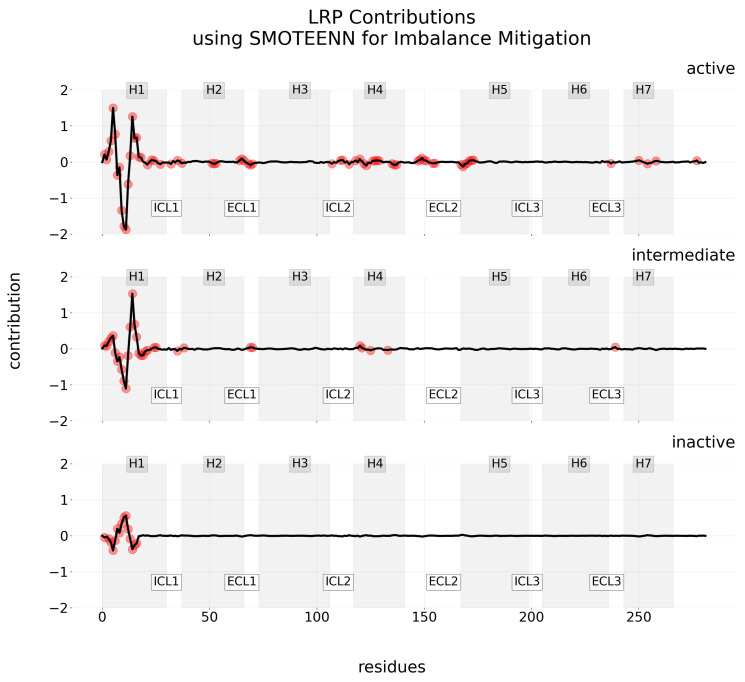
Contribution maps using LRP for interpretability and SMOTEENN as imbalance mitigation technique. Each row visualizes the mean level of contribution (positive or negative) across residues for one of three classes: active, intermediate, and inactive. Red points indicate the most relevant residues in the trajectory, as determined by the IQR. Each of the 7TM regions of the GPCR is identified by a gray-scale background interval. Regions in-between correspond to the extra-cellular loop (ECL) and the intra-cellular loop (ICL).

In the same context, the overall contribution of residues across conformational states is illustrated in Figure 5. For clarity, this figure specifically highlights results from the LRP technique paired with the SMOTEENN imbalance mitigation method, which has demonstrated higher performance. Trough the Interquartile Range (IQR), we have filtered out the most relevant residues—including both negative and positive contributions—and detailed them in Table A1 from Appendix A.

**Figure 6:**
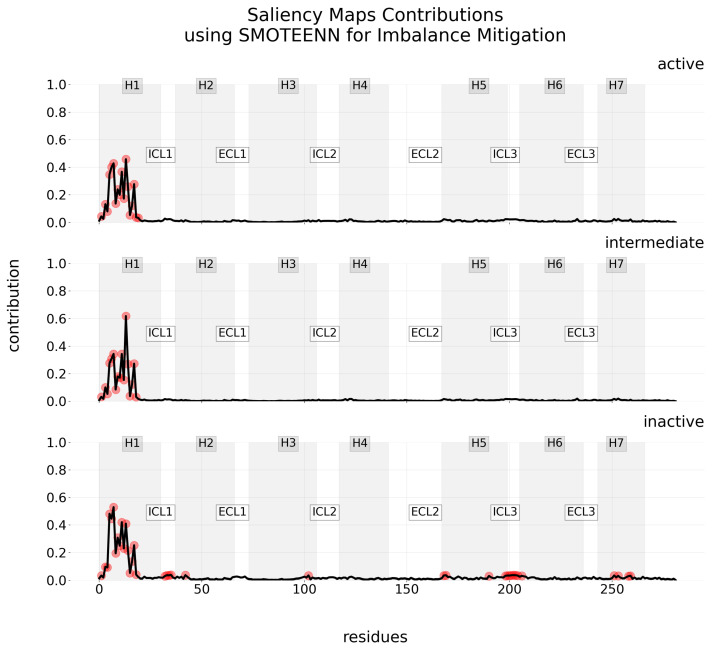
Contribution maps using Saliency Maps for interpretability along with SMOTEENN for mitigating imbalance. Each row visualizes the mean level of contribution—in this case, solely positive contribution—across residues for one of three classes. Red points indicate the most relevant residues as determined by the IQR. The 7TM regions are identified by a gray-scale background, with the regions in between corresponding to ECL and ICL.

In the same vein, a comprehensive compilation of contribution maps for various explainability methods—namely Layer-wise Relevance Propagation (LRP), Local Interpretable Model-agnostic Explanations (LIME), Shapley Additive Explanations (SHAP), and Saliency Maps—under different imbalance mitigation methods are provided in Appendix B. To highlight the unique aspect of Saliency Maps, which compute and display only positive contributions, Figure 6 showcases the contribution maps generated using Saliency Maps when the SMOTEENN imbalance mitigation method is applied.

**Figure 7:**
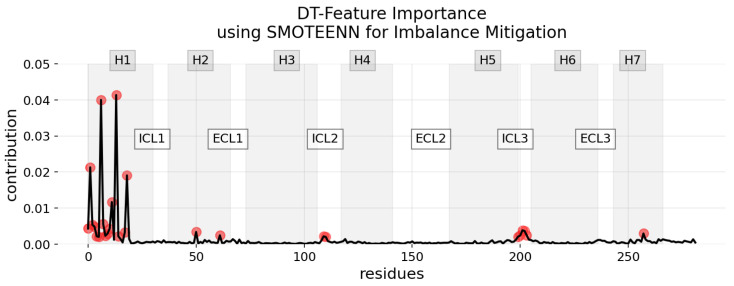
Distribution of feature importance in the DT model with SMOTEENN class imbalance mitigation technique. This plot visualizes the global average contribution of each residue to the model’s decision-making process. Relevant residues, represented as red points, are those with significantly higher or lower importance than the typical range, as determined by the IQR. Gray shaded regions identify the 7TM regions, as in previous figures.

In our results, the feature importance derived from Decision Trees (DT) provides a distinct contrast to the explanations offered by more complex explainability methods, as demonstrated in Figure 7. DTs offer a global interpretation of predictions by identifying key features significant across all classes, rather than delineating specific, class-related attributions of features.

**Figure 8:**
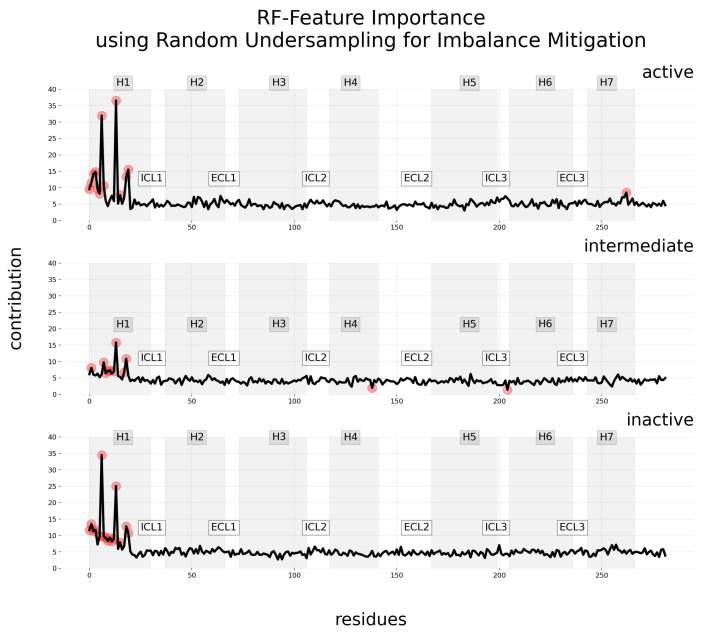
Contribution maps are generated using feature importance with permutation, coupled with the random undersampling technique for addressing data imbalance. Each row in these maps represents the mean contribution level (considering only positive contributions) across residues for one of the three classes: active, intermediate, and inactive. Relevant residues, represented as red points, are those with significantly higher or lower importance than the typical range, as determined by the IQR. The 7TM regions of the GPCR are delineated by varying shades of gray in the background, with regions between them corresponding to ECL and ICL.

The RF variable importance suffers from a similar drawback. Nevertheless, analyzing the reduction in prediction accuracy caused by permuting each feature [59,60] helps to effectively identify which features are most influential in predicting each specific class, as shown in Figure 8. Nevertheless, this method does not reveal negative contributions, which remains a significant drawback.

Concerning the robustness of the explanations, the outcomes of our analyses can be found in Table 2. In turn, Table 3 presents the stability results of various explainable methods associated with the three top-performing models. Results from the simpler models—DT and RF—using feature importance to elucidate predictions are also included.

**Figure 9:**
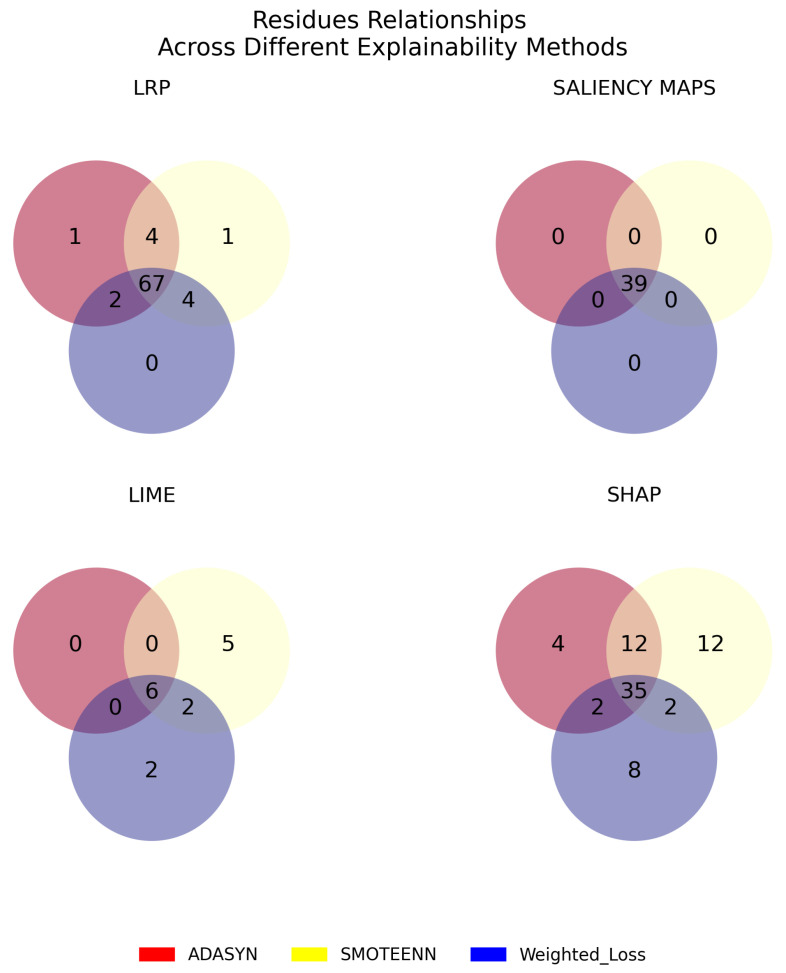
Venn diagrams illustrating the overlap of residues identified as relevant by different explainability methods (LRP, LIME, SHAP, SALIENCY MAPS) for the top-three imbalance correction techniques: ADASYN (red), SMOTEENN (yellow), and Weighted Loss (blue). Each number represents the count of overlapping residues in each scenario.

In assessing the consistency of explanation techniques, Figure 9 features Venn diagrams that denote consensus regarding relevant residues across explainability methods when the top three performing class-imbalance correction methods are applied—namely ADASYN, SMOTEENN, and Weighted Loss. These consensus residues should be the focus of heightened attention in subsequent studies.

Table 4 lists the residues from Venn analysis and identified as significant across all imbalance-mitigation methods using the LRP technique, which appears to be the most consistent method. These residues are named according the Ballesteros–Weinstein numbering system, a standard convention for annotating GPCR structure [61]. Further details about common residues across imbalance methods for the other explainability techniques can be found in Appendix C.

**Figure 10:**
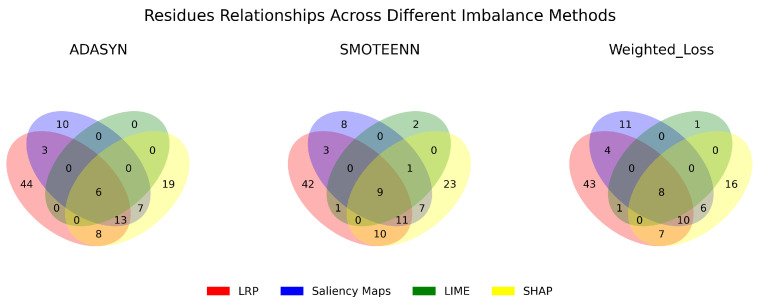
Comparative analysis of residue relationships using different imbalance methods and interpretation predictions techniques.

Correspondingly, Figure 10 presents Venn diagrams that illustrate the level of agreement in identifying relevant residues across the top three best-performing class imbalance mitigation techniques using various interpretability methods. Table 5 lists the residues identified as significant across all methods, representing a consensus among the techniques.

## 3. Discussion

The reported results offer us a broad illustration of the complexities involved in studying the conformational states of the ($\beta_{2}$AR) receptor using a DL method. Here, we discuss the results in terms of performance metrics, highlight consistencies among various methods, and consider the potential implications of our findings for the specific domain of application.

### 3.1. Performance Evaluation

From Figure 2, it is evident that, when the distribution of data is not adjusted, accuracy results can be misleading. This metric can be inflated by a large number of correct predictions for the majority class, resulting in significant bias. SMOTEENN improves precision, recall, and F1-score for minority classes, indicating better handling of class imbalance. While precision and recall offer valuable insights into a classifier’s performance, they have limitations. Recall does not reveal the count of incorrect negative predictions, known as false negatives, and precision does not show how many positive predictions were actually incorrect, referred to as false positives. Additionally, F1-scores inherit the limitations of precision and recall, making it crucial to interpret these metrics within the context of their underlying biases. These limitations are highlighted by the Weighted Loss method’s plot from Figure 2, where it appears to under-perform. This perceived under-performance could stem from an incomplete evaluation that fails to adequately assess the method’s effectiveness in handling class imbalances.

The ROC curves depicted in Figure 1 and the corresponding AUC scores provide us with a quantitative evaluation of the model’s ability to discriminate between conformational states in scenarios of great class imbalance, such as the one posed by the analyzed MD data. Notably, the model struggles to accurately predict the intermediate class, despite its predominance in sample size. Ordinarily, one may expect the trained model to exhibit a clear preference for predicting the majority class. However, the results indicate that the decision boundary between the majority and minority classes is not well-defined. This is because the features discriminating between *active* and *inactive* states are more easily distinguishable from each other than those discriminating either of these states from the *intermediate* state, which hinders achieving high sensitivity.

Nevertheless, the positive impact of resampling techniques is clearly reflected in the results. Techniques that generate synthetic samples, such as SMOTE, ADASYN, and SMOTEENN, demonstrate a more uniform AUC score across all classes, indicating significant improvements in managing class imbalance by creating a more balanced dataset, which in turn enhances the model’s accuracy and fairness by providing a better representation of minority classes. The AUC for the *intermediate* class using SMOTEENN is 0.9616, while ADASYN yields an AUC of 0.9632. In comparison, undersampling-based techniques struggle to discriminate the *intermediate* state, as evidenced, for instance, by the lower AUC score of 0.7804 obtained with the NearMiss algorithm. Random oversampling appears to suffer from the same drawback. Undersampling techniques, while reducing computational load—an inherent drawback of oversampling-based methods—can lead to the loss of crucial information, negatively impacting the model’s performance. By potentially discarding valuable data, undersampling methods risk oversimplifying the problem space, which is particularly detrimental in complex datasets where every sample could carry important insights.

For providing an in-depth evaluation of model performance, the Matthews Correlation Coefficient (MCC) is crucial as it considers all four categories of the confusion matrix, offering a balanced metric unlike simpler measures such as accuracy. MCC’s nuanced view is essential for highlighting discrepancies across classes, which helps prevent bias in models that may otherwise favor over-represented classes. In contrast, ROC curves assess the ability to distinguish between classes but fail to account for class distribution. The significant improvement in MCC scores from the baseline unbalanced dataset to those adjusted with SMOTEENN underscores the effectiveness of this method. SMOTEENN addresses the challenge of accurately predicting underrepresented classes by synthesizing new examples within the minority class and cleaning the data by eliminating outliers, thus preserving data integrity and enhancing model reliability.

Offering a holistic view of model performance is not just beneficial but essential. By integrating the comprehensiveness of MCC with the specificity of ROC curves, we can achieve a more refined analysis of results, ensuring that our models are unbiased and equitable in their predictions, i.e., that the model’s accuracy and reliability are consistent across different groups, not just the majority.

### 3.2. Explainability of the Models

Figure 4 enables us to inspect the results of our methodology with well-known receptor activation mechanisms reported in [58]. The identified residues—ILE121${\;}^{3.40}$, SER204${\;}^{5.43}$, SER207${\;}^{5.46}$, PRO211${\;}^{5.50}$, and PHE282${\;}^{6.44}$—influence significant structural changes in response to agonist binding, thus being crucial in the activation of the receptor. The contribution results from our analysis seem to align with their established roles in the conformational state. For example, SER204${\;}^{5.43}$ shows significant contribution in both *intermediate* and *active* states, confirming its critical role in forming interactions with the agonist that facilitate the receptor’s transition to an active configuration. SER207${\;}^{5.46}$, on the other hand, shows its highest contribution in the *intermediate* state, indicating its involvement in the crucial structural changes during the early stages of activation. PRO211${\;}^{5.50}$ stands out with a clear contribution to the *active* state, suggesting it plays a direct role in stabilizing the receptor’s active conformation. PHE282${\;}^{6.44}$ also shows a notable contribution to the *intermediate* state, reflecting its involvement in the necessary adjustments within the receptor’s core structure that precede full activation. Similarly, ILE121${\;}^{3.40}$ is particularly relevant in the *intermediate* state, indicating its role in the transitional arrangements that help prepare the receptor for activation.

An in-depth analysis of the computed contribution for all residues across explainability methods is included in Appendix B. Each method for providing explanations has strengths and limitations that must be acknowledged. For instance, Layer-wise Relevance Propagation (LRP) can provide detailed, layer-by-layer explanations of the model by elucidating the contribution of each neuron to the final decision. However, interpreting this wealth of information can be challenging and, depending on the problem at hand, unnecessary. Additionally, LRP may become computationally intensive with larger neural networks or datasets. Conversely, LIME, being model-agnostic, offers flexibility and is beneficial for providing straightforward explanations of predictions without the need to understand the model’s internal workings. However, it produces simple, local explanations that may not fully capture the model’s global behaviors, and the reliability of these explanations heavily depends on the perturbations applied. Similarly, SHAP provides comprehensive insights that are consistent and theoretically grounded, yet it also demands significant computational resources, especially for large-scale models. Interestingly, Saliency Maps offer a simpler approach but only account for positive contributions, which may limit their utility in fully understanding the decision-making process.

The feature importance in DT, as shown in Figure 7, exhibits a similar limitation: while it provides a global view of what features are generally important for the model to make any decision, it falls short in offering detailed, class-specific insights. Unlike more granular approaches, feature importance in DT does not differentiate between the nuances that define an active, inactive, or intermediate state, thereby providing a general rather than a granular understanding of the model’s decision-making process. LIME and SHAP can be applied to RF and DT in order to surpass this problem. Nonetheless, RF models are capable of reporting relevant class-specific attributes. In the *caret* package’s RF implementation in R [62], both the feature permutation method and the Gini index approach are incorporated, enhancing its utility in this nuanced class-specific attribute analysis.

From the results of our experimentation, the H1 region is consistently identified as the most significant for classifying conformational states. More specifically, the results in Table A1 from Appendix A reveal that, in the combination of LRP and SMOTEENN, a total of 115 relevant residues were identified to be the most relevant for various conformational states of the receptor, as determined by the IQR. Of these, 60 belong to the H1 region. This finding aligns with previous research [44,63], which also identified the influence of the H1 TM region in a model trained using random undersampling, with explanations generated through LRP. Additionally, in this study, residues from other regions such as ICL1, H2, H4, H5, ECL1, ECL2, and H7 are also shown to contribute in the prediction of conformational states, though in varying degrees and with distinct implications. These results provide detail of the roles these regions play in the receptor’s functionality, even though they may not be as prominently represented as those of H1.

Delving deeper into the results from Table A1, we observe distinct patterns across the active, intermediate, and inactive states. For instance, residues such as VAL34${\;}^{1.33}$, GLY35${\;}^{1.34}$, and MET36${\;}^{1.35}$ show significant positive contributions in the active state, suggesting a pivotal role in receptor activation. Conversely, residues like SER41 and MET40, while showing negative contributions in both active and intermediate states, have positive contributions in the inactive state, suggesting a potential inhibitory role in active states and a facilitating role in the inactive state. Interestingly, the active state has the highest number of unique residues. For example, the positive contribution of residue GLY35${\;}^{1.34}$ may be linked to key interactions or conformational changes necessary for receptor activation, while negative contribution of residue VAL39${\;}^{1.38}$ may suggest a regulatory or conformational counterbalance. Similarly, in the intermediate state, residues such as PHE49${\;}^{1.48}$ and LEU155${\;}^{4.47}$ may provide insights into the structural shifts that occur as the receptor transitions between active and inactive states. Their unique presence in this state underlines their potential role in these transitional dynamics.

### 3.3. Robustness, Stability, and Consistency of the Explanations

In our robustness tests for the 1D-CNN model of different explanation methods under various class-imbalance mitigation techniques—specifically, the top three performers, ADASYN, SMOTEENN, and Weighted Loss—Saliency Maps stood out with superior performance, achieving a near-perfect cosine similarity score of approximately 0.99. However, it is crucial to note that Saliency Maps generate only positive contributions. This limitation can lead to incomplete information, as negative contributions, which are essential for a full understanding of model behaviors, are not represented. Conversely, LIME, which provides a more comprehensive range of contributions, demonstrated the lowest performance in terms of robustness, especially when used with Weighted Loss, where it recorded a cosine similarity score of −0.0407, as detailed in Table 2. This poor performance can be attributed to LIME’s heavy reliance on perturbing the input data, which, when altering the original distribution for testing robustness, may compromise the integrity of the data and lead to potentially less accurate explanations.

In terms of stability, SHAP has proven to be highly stable across repetitions, achieving a perfect cosine similarity score of 1.00, as demonstrated in Table 3 across the top three mitigation methods. This makes it exceptionally effective and reliable in providing explanations. Its theoretical foundation ensures that the contribution of each feature is measured consistently and fairly. Unlike other methods that analyze model decisions, SHAP examines how all features interact to influence the model’s decisions, rather than considering each feature in isolation. However, it is important to note that SHAP is comparatively demanding in terms of computational resources.

It is worth mentioning that LRP, while straightforward and effective in some scenarios, notably underperformed with SMOTEENN and Weighted Loss imbalance mitigation methods. These atypical results suggest that synthetically generated samples from SMOTEENN may not be representative of the data distribution. Additionally, the weighted schema could be distorting the relevance propagation, given that the neural network’s inner workings have been distinctly shaped based on class weights. Meanwhile, LIME remains the worst performer in terms of stability, suggesting its lack of trustworthiness and effectiveness. Notably, while DT and RF may appear more stable, this can be attributed to their inherent design, which involves retraining for each test scenario. This adaptive training approach boosts perceived stability rather than demonstrating an inherent robustness in handling data variations or maintaining consistency across different contexts.

A further assessment of explainability methods has been carried out in terms of consistency, using Venn diagrams in Figure 9 to visually compare and contrast the overlap and divergence among the findings of different methods. For LIME, there is a considerable lack of overlap among the residues when different imbalance methods are applied, suggesting that the generated explanations vary significantly over them, potentially reflecting its vulnerability to slight data shifts. In contrast, LRP has a strong overlap of 67 residues (listed in Table 4) between all three best performing methods (ADASYIN, SMOTEENN, and Weighted Loss). Intriguingly, common residues span among active and intermediate states. This consistency indicates that, despite its under-performance with SMOTEENN and Weighted Loss in robustness and stability metrics, LRP’s explanations remain largely consistent across imbalance-handling techniques.

Similarly, SHAP displayed a good balance, providing consistent explanations across methods. Overall, these results highlight the importance of taking into account both robustness and stability scores and how consistent their explanations are across different imbalance mitigation methods when evaluating how well an explanation method works. Similar analyses have been conducted to determine consistency across class-imbalance correction methods under the various explainability techniques Figure 10. The three best-performing methods, namely ADASYN, SMOTEENN, and Weighted Loss, yield similar insights, identifying six, nine, and eight common residues across explainability methods (Table 5), respectively. In all three cases, these common residues are located within the H1 region during the intermediate state of the protein.

The residues consistently estimated to be relevant from Venn Diagrams, especially around TM region H1, could play a key role in ligand binding, signal transduction, or other receptor functions. The ability to single out these residues with high precision could guide future experimental designs, potentially leading to the development of more targeted therapeutics or better understanding of receptor dynamics. Moreover, having a clearer understanding of the conformational states of the $\beta_{2}$AR receptor and the residues that play crucial roles in these states can help predict receptor behavior in response to different ligands or external stimuli.

## 4. Materials and Methods

We base our study on MD simulation data obtained by Kohlhoff et al., on the Google Exacycle platform, details of which can be found in [43]. The accumulated simulation time is 2.15 ms, produced by strategically employing multiple short parallel simulations, each culminating in distinct rounds that, when combined via Markov state models (MSMs), offered a nuanced description of the GPCR activation landscape. According to [43], running numerous shorter trajectories increases the probability of capturing rare and biologically significant events, such as the elusive transient intermediate states critical to GPCR function. These intermediate states are often overlooked in the continuum of longer, singular simulations due to their transient nature.

**Figure 11:**
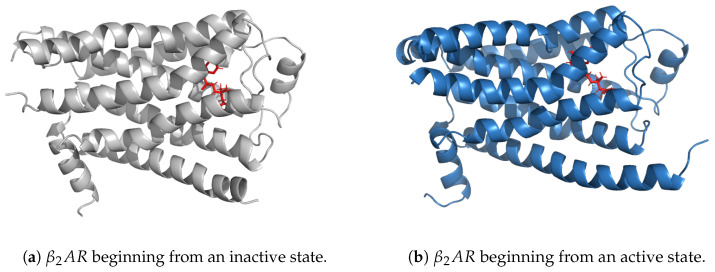
Crystal structure model of the β2-adrenergic receptor with the full agonist BI-167107 in red. The image provides a detailed three-dimensional portrayal of the receptor in two conformations: initiated from an inactive state (**a**) and from an active state (**b**).

A molecular visualization of the $\beta_{2}$AR receptor bound to the agonist BI-167107 is shown in Figure 11.

The dataset encompasses simulations of both the inactive (PDB 2RH1) and active (PDB 3P0G) crystal structures of the $\beta_{2}$AR receptor, employing two specific ligands: the partial inverse agonist Carazolol and the full agonist BI-167107. Additionally, simulations without ligands are included to provide a baseline for understanding ligand-independent receptor behavior. These simulations are designed to elucidate sequences of conformational transformations and interactions that a receptor undergoes as it transitions between different states—active, inactive conformations and meta-stable intermediate states—in response to binding with specific ligands. By starting from the inactive state and introducing the full agonist, we observe the transition toward activation, revealing the conformational changes necessary for receptor activation. Conversely, simulations beginning with the active state and introducing the inverse agonist explore the potential pathways leading back to the inactive conformation.

In our case study, we aim to elucidate key motifs, specifically crucial residues and their roles in the conformational transformations during the receptor’s transition between states. For this, the explanations from a DL-based model serve as a relevant tool in the knowledge generation realm. We concentrate on establishing a robust analytical framework with a particular focus on the inactive structure of the $\beta_{2}$AR receptor bound to the full agonist BI-167107, anticipating that these results can be readily extrapolated to analyze other GPCR structures.

### 4.1. Data Pre-Processing

In our approach, we transformed the high-dimensional molecular dynamics (MD) data using both feature engineering and dimensionality reduction. We simplified the structural information of the receptor by calculating the center of mass of each residue from its constituent atoms in every simulation sample. This method not only reduces data dimensionality while preserving the overall movement of the residues but also provides clearer insights into their dynamics, which are vital for deciphering complex structural shifts. For simplicity, each trajectory is represented as single 846-dimensional vector (282 residues multiplied by 3 coordinates) rather than processing each coordinate separately. This unified representation helps us maintain the spatial relationships between residues and makes feature extraction more straightforward in our subsequent analysis.

In describing the receptor states, the focus is placed on the distance between specific helices in the receptor structure, as described in [43]. In particular, we have considered the distance between the transmembrane helix 3 (H3) and helix 6 (H6), measured using the alpha-carbon atoms of residues arginine at position 131 and leucine at position 272 of the amino acid sequence, abbreviated as R131 and L272, respectively. This metric is acknowledged as one of the experimentally well-known features of GPCRs associated with activation states [16,64]. A significant change in the H3–H6 distance not only serves as an indicator of the receptor’s conformational transitions, but it is also significant for the receptor’s interaction with other proteins, which in turn, could impact signaling pathways. In the receptor, the H3–H6 distances in the *active* and *inactive* conditions are, in turn, higher than or equal to 14 Å, or lower than or equal to 8.5 Å; otherwise, the state is considered to be *intermediate*. While global metrics like the root mean square deviation (RMSD) of atomic positions offer a comprehensive view of structural deviations, the simplicity and directness of measuring the distance between two distinct points such as residues R131 and L272 provides an immediate and interpretable insight into the receptor’s structural shifts.

### 4.2. Class Imbalance

**Figure 12:**
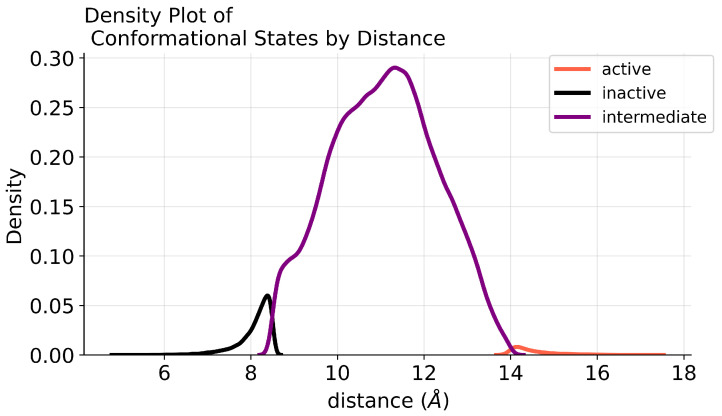
Density plot showcasing the distribution of conformational states (Active, Intermediate, Inactive) across varying distances (in Angstroms). It illustrates the intricacy in distinguishing between inactive and active conditions, as they manifest within a narrower range during the transition, occurring gradually until they become evident.

The dataset under study exhibits a significant class imbalance, with the intermediate state being heavily predominant, indicating a tendency of the receptor to linger in transitional—intermediate—conditions. The density plot in Figure 12 visually highlights the distribution characteristics of the three classes. The intermediate state class is widely spread, taking a broader range of values. In contrast, the sharper peaks observed for the active and inactive states reflect a narrower distribution with higher frequencies around specific values. This imbalance can lead to biased model predictions, favoring the majority class (in this case, the intermediate state) in detriment of the minority classes (active and inactive states).

**Figure 13:**
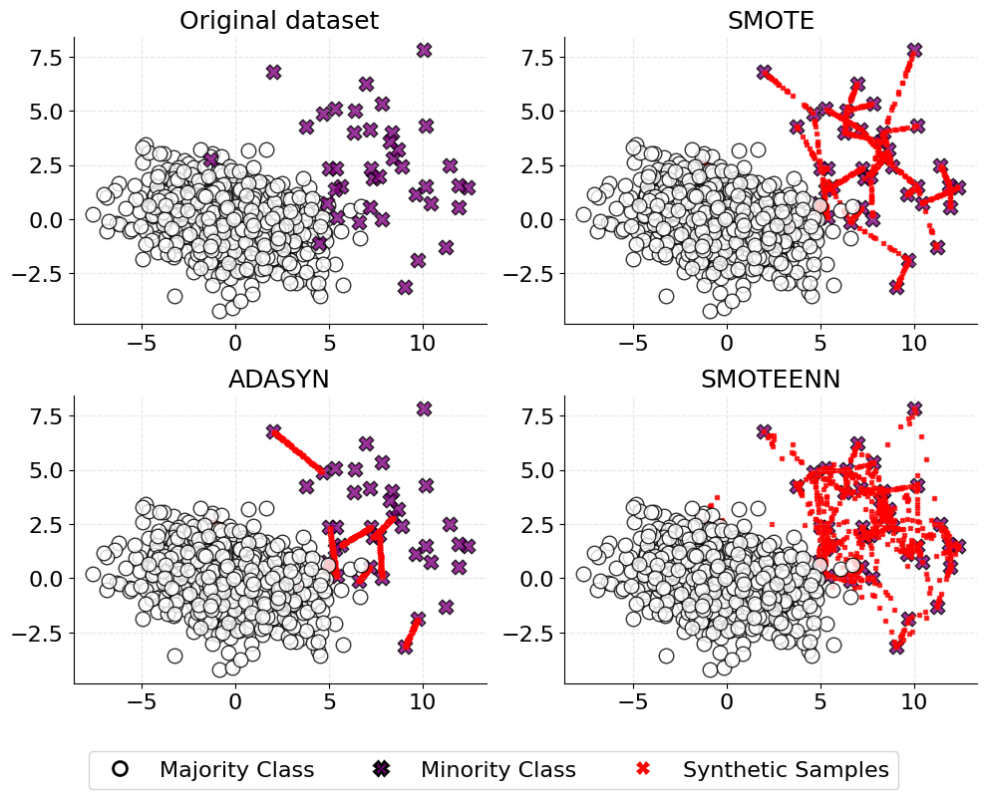
This figure illustrates how SMOTE, ADASYN, and SMOTEENN techniques address class imbalance in a 2D feature space. The original dataset (**top-left image**) shows the minority class (purple X’s) significantly underrepresented compared to the majority class (white circles). SMOTE (**top-right image**) generates new minority instances along the lines connecting existing points. ADASYN (**bottom-left image**) focuses on areas near the decision boundary to create synthetic samples, resulting in a more varied distribution of the minority class. SMOTEENN (**bottom-right image**) combines over-sampling with under-sampling, cleaning the synthetic samples by removing those that are classified as noise or are in regions of class overlap. Synthetic data points are depicted in red.

In our previous work, we rigorously addressed the issue of dataset imbalance by experimenting with various re-sampling techniques [65]. Both the undersampling of the majority class and the oversampling of the minority class were explored, employing methods such as ADASYN (Adaptive Synthetic [66]), SMOTE (Synthetic Minority Over-sampling Technique [67]), SMOTEENN (SMOTE + Edited Nearest Neighbors [68]), NearMiss Algorithm, Random Oversampling, and Random Undersampling to ensure a more evenly distributed representation of the conformational states. SMOTE and ADASYN are popular oversampling methods that generate synthetic samples of the minority class; they are illustrated in Figure 13.

SMOTE creates synthetic samples by finding the k-nearest-neighbors for minority class observations and drawing a line between the neighbors, then generating random points along these lines [67,69]. ADASYN, instead, adapts the density distributions of the minority class to create synthetic data [66]. While both methods enhance the minority class representation, they may create overlapping classes if the synthetic samples are generated without considering the majority class distribution, potentially leading to increased false positives. In the same line, SMOTEENN (bottom-right image in Figure 13) combines over-sampling of the minority class via SMOTE with cleaning of the majority class by removing any majority samples whose class label differs from at least two of its three nearest neighbors [67,68,70,71,72]. This hybrid method attempts to provide a well-balanced dataset, but it may become computationally intensive as the dataset size increases.

Random Oversampling and Random Undersampling are simpler techniques, wherein the former duplicates random instances of the minority class, and the latter removes random instances of the majority class to achieve a more balanced dataset [73,74]. While these methods are computationally less demanding, they can either exacerbate overfitting (in the case of Random Oversampling) or lose potentially crucial information (in the case of Random Undersampling). In a similar vein, the NearMiss Algorithm is an undersampling technique, which selects examples based on the distance to the nearest examples for the majority class, aiming to provide a balance between the classes. While this technique is as straightforward to implement as Random Undersampling, there is a risk of discarding crucial data. Lastly, the Weighted Loss method was assessed, which, rather than altering the original data distribution, adjusts the cost function to penalize miss-classifications of the minority class more severely, as seen in refs. [75,76,77]. This offers the advantage of training on the genuine dataset without the need for synthetic samples or removing existing ones. The weighting scheme for each class is calculated by taking the total number of instances ($n_{c}$) in the dataset and dividing it by the number of instances in a particular class ($n_{t}$), $1-\frac{n_{c}}{n_{t}}$. This ratio represents the class imbalance compared to the overall dataset.

In analyzing these techniques, it becomes evident that there is a trade-off between achieving a balanced dataset, maintaining the original data information, and computational efficiency. The selection of the most appropriate method is problem-dependent [78,79]. In this context, the correct assessment of the performance of our post-resampling classification model becomes crucial for generating a better understanding of the generalization across all classes. A diverse set of metrics is used in our experiments to provide robust insights into the classification performance.

The Confusion Matrix is a key tool for assessing the model predictions, also serving as the source from which several relevant performance metrics can be derived. The Accuracy metric provides a general understanding of the model predictions, but it is inadequate for imbalanced scenarios. A model could show high accuracy by merely predicting the majority class, thus entirely missing out on the minority class instances. Here, the Precision metric provided a measurement into the model’s exactness when making positive predictions. Nevertheless, it does not inform about the model’s ability for identifying all actual positive instances, a gap filled by the Recall metric. While these metrics together provide a more comprehensive look at the model performance, they exclusively focus on positive instances, overlooking true negatives which can be crucial depending on the specific domain or problem at hand [80]. The F1-score, a blend of Precision and Recall, aims at enhancing the model performance measurement, yet still leans heavily on true negatives.

The MCC is a more informative measurement in imbalanced scenarios. Unlike the other metrics, MCC considers all quadrants of the confusion matrix—true positives, true negatives, false positives, and false negatives, thus offering a balanced view of the performance across all classes. MCC values range from −1 to +1, with +1 describing perfect prediction, 0 indicating random prediction, and −1 representing inverse prediction.

### 4.3. XAI Methods

In our previous research, we extensively explored the explanations of the predictions made by the DL model using the LRP method under various class imbalance mitigation techniques [65]. Our aim was to ensure that the unveiled information was unbiased and did not predominantly reflect the characteristics of the majority class (the intermediate condition of the receptor). LRP provides a granular view of how different conformational states are perceived, by decomposing the prediction into contributions from individual features [81]. Initially, the prediction is transferred from the output layer to the input layer. As it moves backward trough the layers, the so-called relevance (the contribution to the final prediction) of each neuron to the preceding ones is redistributed based on the contribution of individual neurons; a neuron that contributes more receives a larger share of the relevance. Various so-called rules exist for propagating the relevance values back through the network for providing a fine-grained explanation. Once the relevance has been distributed throughout the entire network, it is possible to generate a visualization in form of a heatmap. Such visualization aids to emphasize the contribution of each input feature to the final prediction.

Although LRP is a valuable asset for unveiling relevant information within the conformational space, the exploration of multiple explanation methods could yield a more comprehensive understanding of the problem and it may increase the trustworthiness of the results [82]. Additionally, finding consistency across different explanation methods can increase the confidence in the obtained explanations and the insights derived from them. For the experiments reported in this paper, we have relied on common explanation-generating methods such as LIME [82], SHAP [83], and Saliency Maps [84], alongside LRP.

Saliency Maps and LRP are specifically designed for elucidating the predictions of neural network models. Saliency Maps are created by computing the gradient of the prediction with respect to the input data. This gradient, computed using back-propagation, indicates how the output value changes with small changes in the input. In essence, the gradients tell us which features need to be changed the least to affect the model’s decision the most. The primary advantage of Saliency Maps resides in their simplicity and the direct visual explanation they provide. However, these maps tend to emphasize features broadly rather than in a granular approach. Additionally, they are non-negative, i.e., they display regions of positive contribution to the model’s decision, without representing inhibitory or negative effects.

**Figure 14:**
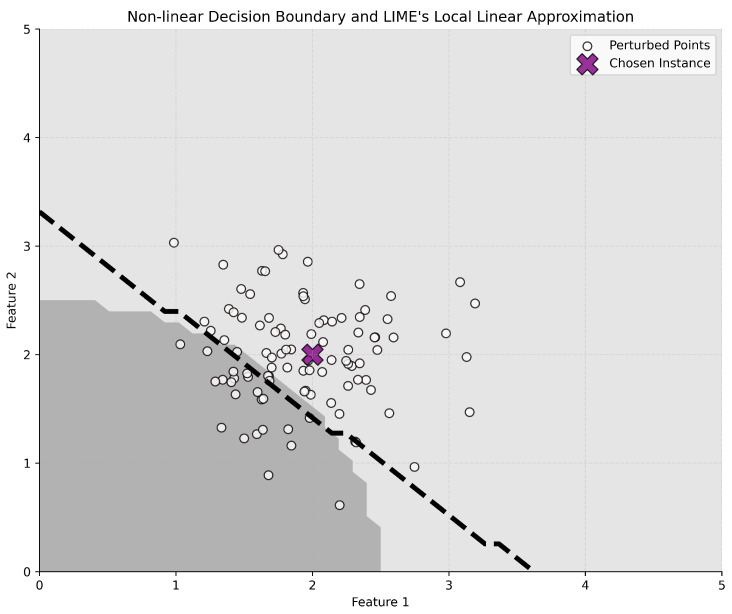
Illustration of how LIME approximates the nonlinear decision boundary of a model (delineated by shades of gray) with a simpler, linear boundary (dashed black line) in the vicinity of a specific data point. The white dots are the perturbed data points sampled around the chosen instance, which is marked in purple.

On the other hand, LIME and SHAP are model-agnostic methods that have demonstrated broader applicability across different types of ML models. LIME operates by examining individual data points. It introduces slight perturbations to these points, creating a set of subtly altered instances. These perturbed instances are then weighted based on their proximity to the original data point. Using this weighted dataset, LIME trains surrogate models—often linear regression models—to approximate the behavior of the more complex model, as illustrated in Figure 14.

By dissecting these surrogate models, LIME discerns the relative importance of each feature in the predictions, offering interpretable insights into the complex model’s local decision-making process, although at the cost of the computational overhead of repeatedly constructing these local surrogate models. Unlike LIME, SHAP does not rely on surrogate models; instead, it uses principles from game theory to compare what a model predicts with and without each feature, across many different combinations of features, to calculate each feature’s contribution to the prediction. While SHAP provides clear insights into the model’s behavior, it can be slow and requires a significant amount of computing power, especially for models with many features, complex structures or many samples—produced by the usage of oversampling methods—if seeking a global explanation. Along with the computational demands, we are also faced with the subjectivity involved in selecting neighborhoods and other parameters specific to these techniques [85,86].

Each of the methods described in the previous paragraphs has relative strengths, making them well-suited to different tasks depending on the need for model-specific or model-agnostic explanations, local or global insights, and visual or quantitative explanations. In this context, we endeavor for an unified approach that would allow us to capture a broader/global understanding of the predictions, while also giving due consideration to explaining specific features that play a pivotal role in shaping the conformational conditions of the receptor. Additionally, our objective is to generate both visual and quantitative analyses of the explanations produced, extending the application to one-dimensional data analysis derived from MD simulations.

### 4.4. Assessment of Explanation Methods

In the field of XAI, the robustness and stability of predictions are not merely advantageous but essential. These qualities ensure that the explanations provided by AI models are not only accurate under controlled conditions but also remain consistent across varying environments and datasets [87,88]. This consistency is vital for generalizability, ensuring that the insights gained from one set of data can be effectively applied to another without loss of fidelity. Robust explanations can withstand minor perturbations in input data, which is crucial in dynamic real-world applications where data variability is common. Similarly, stable explanations guarantee that the interpretative outcomes are repeatable and reliable over time, providing a dependable basis for decision-making. Although a wide range of explainability methods exists, assessing the quality and trustworthiness of these explanations remains an open challenge. Several criteria have been proposed for this purpose. For instance, one study measures the fidelity of LRP-produced explanations by estimating the drop in prediction score resulting from the deletion of pixels deemed important by an explanation method [89]. Similarly, another source highlights how perturbations affect the quality of explanations and their practical application in real-world scenarios [90].

We assess the robustness and stability of distinct explanation techniques under different imbalance methods drawing inspiration from [57]. For assessing robustness, we perturbed the inputs using Gaussian noise with a zero mean and a standard deviation of 0.5, ensuring that the distribution of the data is not substantially altered while generating random noise that could affect the explanations. Using this approach, we validate whether the features identified as important by the different explainability techniques substantially impact the model outputs. Likewise, the assessment of stability is carried out by randomly selecting a representative population from the dataset and randomly repeating the explanations between two to five times to assess whether the results remain consistent. In our study, the consistency of explanations, both in terms of robustness and stability, is evaluated using the cosine similarity. This metric computes the cosine of the angle between two non-zero vectors, with its values ranging between −1 and 1. A higher value (closer to 1) indicates that the explanations are more similar concerning the orientation of these vectors, i.e., they are directionally aligned. This alignment can signify that the explanations consistently identify the same features as important, maintaining the relative importance of these features across different instances or conditions. Thus, using this assessment, we can ascertain whether different explanation methods in varying settings yield consistent interpretations of the model’s behavior. Broadly, by employing input perturbations and repetition across a representative population, our methodology enables a thorough assessment of explanation consistency against potential bias introduced by imbalanced data.

### 4.5. Experimental Setup

In this section, we detail the experimental setup for the investigation of the conformational space of the $\beta_{2}AR$ receptor. The methodology builds upon previous work, where we addressed a multi-class classification problem using DL-based models to discriminate between active, intermediate, and inactive conformations [65]. Broadly, the process can be broken down into several phases.

Initially, MD simulations were transformed into a suitable format to facilitate analysis, relying on the computation of the center of mass to produce 1D vectors, as previously mentioned. Data were partitioned into training and validation sets to ensure model generalization. These data were then fed into a 1D-CNN model architecture tested across different configurations and enriched with distinct class imbalance mitigation methods. Our 1D-CNN model was methodically developed layer-by-layer, drawing inspiration from an empirical strategy emphasizing iterative assessments of various configurations and evaluation metrics, as detailed in [65]. Performance evaluation used metrics suitable for the imbalanced scenario, including Precision, Recall, F1-score, and particularly the MCC score.

In addition, we computed traditional Receiver Operating Characteristic (ROC) curves using a One-vs-All (OvA) approach to address our multi-class problem. In the same vein, we benchmarked the results obtained from the 1D-CNN against simple classifiers such as Decision Trees (DT) and Random Forests (RF), considering feature importance analysis to elucidate their predictions. This may be seen as a baseline performance comparison with DL, but also from the point of view that classifiers such as DT and RF are more readily interpretable by design.

As previously described, several XAI methods, including LRP, LIME, SHAP, and Saliency Maps, were utilized. Each method produced a contribution map that emphasized the importance of motifs for each receptor condition. The analysis was further refined using the IQR. This approach helps identify cases often overlooked as outliers but yet significant as the most prominent residues in our study. Lastly, the evaluation of the robustness and stability of XAI techniques concentrated on the top three performing models: SMOTEENN, ADASYN, and Weighted Loss. Furthermore, we conducted a consistency analysis using Venn diagrams to reveal overlapping residues across explainability techniques under the best-performing imbalance methods.

## 5. Conclusions

In this study, we explored the conformational dynamics of the $\beta_{2}$AR receptor from MD simulations using ML models. Ensuring that the natural class-imbalance of these data in terms of their conformational states adscription does not bias the ML models’ performance is paramount. In turn, ensuring that black-box ML models’ predictions are explainable is crucial. Dealing with both potential limitations in parallel ensures the robustness of the predictions and their trustworthiness. This approach guarantees that the findings are not influenced by anomalies or unrepresentative features. If the model’s predictions are biased, the explanations may mistakenly emphasize irrelevant features, leading to inaccurate models and, consequently, to incorrect conclusions. Robust explanations ensure that the outcomes generated are minimally affected by minor fluctuations, enhancing generalization and building trust. Stability means that the outcomes do not vary significantly from one case to another unless the data themselves are substantially different.

In our experiments, we address the explainability of our 1D-CNN ML model through various post hoc techniques, both model-specific and model-agnostic. Each selected method is tailored to match the demands of our study, balancing the need for explainability, computational efficiency, and depth of insight into the model’s behavior. For our study case, a deep dive into the internal mechanics of our model is not as crucial as providing clear, fast, and human-readable explanations of the model’s decisions. These explanations helped us in constructing a detailed analysis to observe distinctive patterns associated with different conformational states predicted by our model in the form of contribution maps. In this context, the LRP explainability technique has been shown to offer a straightforward method for generating clear and consistent explanations, particularly useful in scenarios wherein model-agnostic explanations are not required. Conversely, the LIME method appears to be less effective in the reported evaluations, including robustness, stability, and consistency. Notably, the SHAP technique delivers remarkable performance at the cost of high computational demand.

By correcting for class imbalance, the model’s ability to generalize from a limited number of samples is significantly enhanced. This ensures that insights drawn from the data are not biased toward the majority class, leading to more accurate and reliable interpretations of the receptor’s conformational states. Likewise, an accurate evaluation of classification results is crucial, as an incorrect assessment could depict an overly optimistic picture of the model’s performance, particularly in problems characterized by severe skewed distributions among classes. The systematic assessment of classification results is crucial for reinforcing confidence in the model’s performance while avoiding biases. This entails employing a variety of metrics and benchmarking against established baselines, in order to ensure that the techniques employed genuinely enhance classification performance without introducing bias. When class imbalance is not treated, the MCC scores were markedly lower, indicating poorer generalization and biased predictions.

Importantly, the decision to calculate the center of mass for each residue represents a strategic approach to simplify and clarify the MD data. Effective data processing and transformations are both essential for preparing the dataset for ML applications, ensuring that the inherent challenges of working with high-dimensional data are managed effectively. By focusing on the center of mass, we distill complex atomic coordinates into a more manageable form that captures the essential movements and interactions within the receptor. This method not only reduces the data dimensionality, but also preserves crucial information about the dynamics of the molecular structure, facilitating a deeper understanding of the receptor’s behavior. Furthermore, following the results of Kohlhoff et al. [43], we have established a labeling method that categorizes the receptor states based on the distance between specific helices, thereby enhancing the interpretability of the MD simulations. This targeted approach allows us to directly relate structural changes to functional outcomes, such as activation states, providing clear insights into the mechanisms that govern receptor activity. Accurate data treatment not only ensures the reliability of model predictions but also enhances the clarity and applicability of the explanations, thereby directly influencing the success of downstream applications and research outcomes.

Broadly, the insights about relevant motifs obtained in our analyses may become significant in pharmacoproteomics by enhancing our understanding of the inner mechanisms of receptors. Each conformational state of a GPCR can be associated with distinct functional outcomes, influencing how the receptor interacts with ligands. For example, the active conformational state of a GPCR may be associated with agonist binding and signal transduction, leading to a physiological response. Conversely, the inactive state may be targeted by antagonists to prevent unwanted signaling.

Lastly, the proposed analytical methodology has considerable potential for expansion. This could involve exploring other GPCRs, investigating alternative DL architectures, or integrating experimental data that encompass a broader range of conditions. Such advancements could greatly enhance the predictive capabilities of our models and effectively bridge the gap between computational models and actual biological systems.

## Figures and Tables

**Table 1:** Weighted average metrics of classification results for 1D-CNN, RF and DT, with SMOTEENN imbalance mitigation method.

Model	Precision	Recall	F1-Score
1D-CNN	0.915593	0.906689	0.908289
DT	0.710800	0.574343	0.580146
RF	0.685715	0.564699	0.484628

**Table 2:** Summary of robustness results using cosine similarity. Best results in bold.

Model	Explainable Method	Imbalance Method	Cosine Similarity
1D-CNN	Saliency Maps	ADASYN	0.9882
1D-CNN	Saliency Maps	SMOTEENN	**0.9884**
1D-CNN	Saliency Maps	Weighted Loss	**0.9884**
1D-CNN	LRP	ADASYN	**0.5306**
1D-CNN	LRP	SMOTEENN	0.0296
1D-CNN	LRP	Weighted Loss	−0.0565
1D-CNN	LIME	ADASYN	0.0217
1D-CNN	LIME	SMOTEENN	**0.03145**
1D-CNN	LIME	Weighted Loss	−0.0407
1D-CNN	SHAP	ADASYN	0.3126
1D-CNN	SHAP	SMOTEENN	0.2870
1D-CNN	SHAP	Weighted Loss	**0.3225**
DT	Feature Importance	ADASYN	**0.3605**
DT	Feature Importance	SMOTEEN	0.3016
RF	Feature Importance	ADASYN	0.8704
RF	Feature Importance	SMOTEEN	**0.8953**

**Table 3:** Summary of stability results using cosine similarity. Best results in bold.

Model	Explainable Method	Imbalance Method	Cosine Similarity
1D-CNN	Saliency Maps	ADASYN	0.9531
1D-CNN	Saliency Maps	SMOTEENN	0.9506
1D-CNN	Saliency Maps	Weighted Loss	**0.9545**
1D-CNN	LRP	ADASYN	0.6550
1D-CNN	LRP	SMOTEENN	0.6393
1D-CNN	LRP	Weighted Loss	**0.6649**
1D-CNN	LIME	ADASYN	**0.6172**
1D-CNN	LIME	SMOTEENN	0.5908
1D-CNN	LIME	Weighted Loss	0.5772
1D-CNN	SHAP	ADASYN	**1.0000**
1D-CNN	SHAP	SMOTEENN	**1.0000**
1D-CNN	SHAP	Weighted Loss	**1.0000**
DT	Feature Importance	ADASYN	**1.0000**
DT	Feature Importance	SMOTEEN	**1.0000**
RF	Feature Importance	ADASYN	**1.0000**
RF	Feature Importance	SMOTEEN	**1.0000**

**Table 4:** Relevant residues (as they consistently influence different conformational states) overlapping across imbalance methods when using LRP. First column: conformational state of the residue; second column: residue identifier; third column: residue’s transmembrane helix identifier; fourth column: computed average contribution of the residue.

State	Residue Name	Transmembrane	Mean Contribution
active	ASN51${\;}^{1.50}$	H1	−0.0649
active	LEU53${\;}^{1.52}$	H1	0.0566
active	ALA57${\;}^{1.56}$	H1	−0.0640
active	GLU62	ICL1	−0.0594
active	VAL67${\;}^{2.38}$	H2	−0.0392
active	MET82${\;}^{2.53}$	H2	−0.0418
active	ILE94${\;}^{2.65}$	H2	0.0419
active	LEU95${\;}^{2.66}$	H2	0.0848
active	MET96${\;}^{2.67}$	H2	0.0423
active	MET98	ECL1	−0.0461
active	SER137	ICL2	−0.0380
active	TYR141	ICL2	0.0388
active	GLN142	ICL2	0.0579
active	LEU145	ICL2	−0.0624
active	ASN148${\;}^{4.40}$	H4	0.0428
active	VAL152${\;}^{4.44}$	H4	−0.0462
active	ILE153${\;}^{4.45}$	H4	−0.0865
active	MET156${\;}^{4.48}$	H4	0.0341
active	VAL157${\;}^{4.49}$	H4	0.0462
active	TRP158${\;}^{4.50}$	H4	0.0440
active	ILE159${\;}^{4.51}$	H4	0.0389
active	SER165${\;}^{4.57}$	H4	−0.0547
active	PHE166${\;}^{4.58}$	H4	−0.0610
active	LEU167${\;}^{4.59}$	H4	−0.0669
active	HIS178	ECL2	0.0518
active	GLN179	ECL2	0.1001
active	GLU180	ECL2	0.0514
active	GLN197${\;}^{5.36}$	H5	−0.0591
active	ALA198${\;}^{5.37}$	H5	−0.1251
active	TYR199${\;}^{5.38}$	H5	−0.0477
active	ALA200${\;}^{5.39}$	H5	−0.0334
active	ILE201${\;}^{5.40}$	H5	0.0375
active	ALA202${\;}^{5.41}$	H5	0.0496
active	SER203${\;}^{5.42}$	H5	0.0515
active	GLN299	ECL3	−0.0319
active	ASN312${\;}^{7.39}$	H7	0.0451
active	TYR316${\;}^{7.43}$	H7	−0.0420
active	LEU339	C-Terminus	0.0428
intermediate	VAL31${\;}^{1.30}$	H1	0.0926
intermediate	TRP32${\;}^{1.31}$	H1	0.0788
intermediate	VAL33${\;}^{1.32}$	H1	0.2100
intermediate	VAL34${\;}^{1.33}$	H1	0.2947
intermediate	GLY35${\;}^{1.34}$	H1	0.3588
intermediate	MET36${\;}^{1.35}$	H1	−0.1127
intermediate	GLY37${\;}^{1.36}$	H1	−0.3433
intermediate	ILE38${\;}^{1.37}$	H1	−0.2234
intermediate	VAL39${\;}^{1.38}$	H1	−0.5593
intermediate	MET40${\;}^{1.39}$	H1	−0.8689
intermediate	SER41${\;}^{1.40}$	H1	−1.0831
intermediate	LEU42${\;}^{1.41}$	H1	−0.1794
intermediate	ILE43${\;}^{1.42}$	H1	0.6054
intermediate	VAL44${\;}^{1.43}$	H1	1.5149
intermediate	LEU45${\;}^{1.44}$	H1	0.6725
intermediate	ALA46${\;}^{1.45}$	H1	0.3262
intermediate	ILE47${\;}^{1.46}$	H1	−0.1183
intermediate	VAL48${\;}^{1.47}$	H1	−0.1742
intermediate	PHE49${\;}^{1.48}$	H1	−0.1774
intermediate	GLY50${\;}^{1.49}$	H1	−0.0678
intermediate	VAL54${\;}^{1.53}$	H1	0.0348
intermediate	ILE55${\;}^{1.54}$	H1	0.0429
intermediate	GLN65	ICL1	−0.0533
intermediate	THR68${\;}^{2.39}$	H2	0.0326
intermediate	TRP99	ECL1	0.0437
intermediate	THR100	ECL1	0.0389
intermediate	ALA150${\;}^{4.42}$	H4	0.0857
intermediate	LEU155${\;}^{4.47}$	H4	−0.0386
intermediate	ASN301	ECL3	0.0493

**Table 5:** Overlapping relevant residues across different explainability methods when using distinct imbalance methods. First column: explainability method; second column: state of the residue; third column: residue identifier; fourth column: residue’s transmembrane helix identifier; fifth column: computed average contribution of the residue.

Explainability	State	Residue	Transmembrane	Mean Contribution
ADASYN	intermediate	ILE43${\;}^{1.42}$	H1	0.5594
ADASYN	intermediate	GLY37${\;}^{1.36}$	H1	−0.1150
ADASYN	intermediate	MET36${\;}^{1.35}$	H1	0.0144
ADASYN	intermediate	LEU42${\;}^{1.41}$	H1	−0.0936
ADASYN	intermediate	ILE47${\;}^{1.46}$	H1	−0.1187
ADASYN	intermediate	VAL39${\;}^{1.38}$	H1	−0.0259
SMOTEENN	intermediate	ILE43${\;}^{1.42}$	H1	0.4544
SMOTEENN	intermediate	SER41${\;}^{1.40}$	H1	−0.1997
SMOTEENN	intermediate	GLY37${\;}^{1.36}$	H1	−0.1156
SMOTEENN	intermediate	MET36${\;}^{1.35}$	H1	0.1013
SMOTEENN	intermediate	LEU42${\;}^{1.41}$	H1	−0.0684
SMOTEENN	intermediate	VAL34${\;}^{1.33}$	H1	0.0175
SMOTEENN	intermediate	ILE47${\;}^{1.46}$	H1	−0.0856
SMOTEENN	intermediate	VAL39${\;}^{1.38}$	H1	−0.0130
SMOTEENN	intermediate	VAL48${\;}^{1.47}$	H1	0.0131
Weighted Loss	intermediate	ILE43${\;}^{1.42}$	H1	0.1535
Weighted Loss	intermediate	SER41${\;}^{1.40}$	H1	−0.1194
Weighted Loss	intermediate	GLY37${\;}^{1.36}$	H1	−0.1190
Weighted Loss	intermediate	MET36${\;}^{1.35}$	H1	0.0838
Weighted Loss	intermediate	LEU42${\;}^{1.41}$	H1	−0.0501
Weighted Loss	active	ILE47${\;}^{1.46}$	H1	0.3491
Weighted Loss	intermediate	VAL39${\;}^{1.38}$	H1	0.0416
Weighted Loss	intermediate	VAL48${\;}^{1.47}$	H1	0.0164

**Figure A1:**
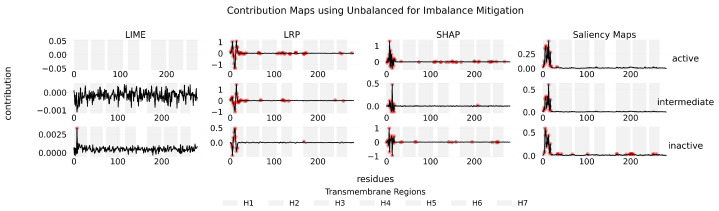
Contribution maps over an unbalanced distribution. Relevant residues are represented as red points.

**Figure A2:**
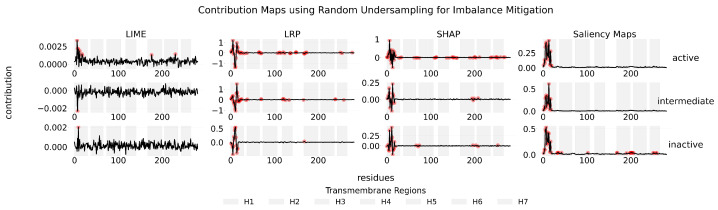
Contribution maps under Random Undersampling. Relevant residues are represented as red points.

**Figure A3:**
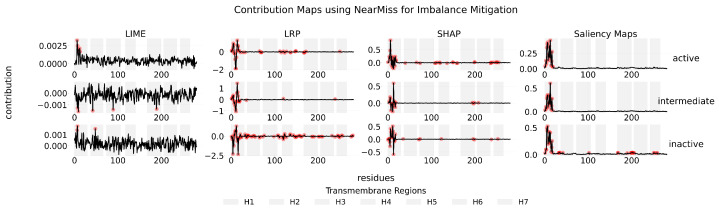
Contribution maps under Near-Miss algorithm. Relevant residues are represented as red points.

**Figure A4:**
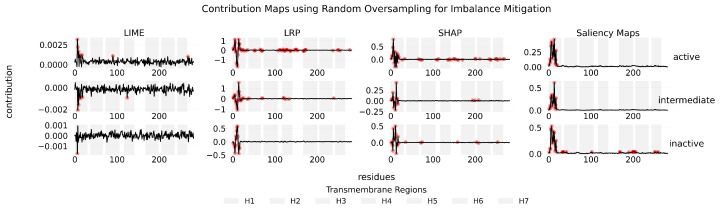
Contribution maps under Random Oversampling. Relevant residues are represented as red points.

**Figure A5:**
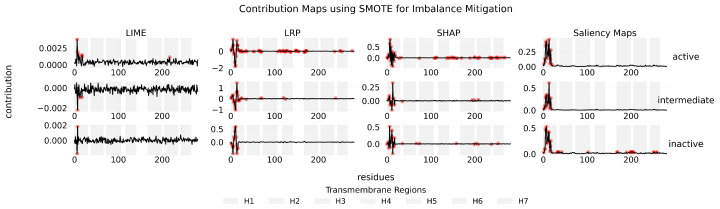
Contribution maps under SMOTE. Relevant residues are represented as red points.

**Figure A6:**
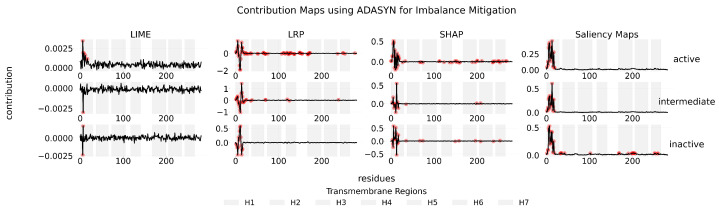
Contribution maps under ADASYN. Relevant residues are represented as red points.

**Figure A7:**
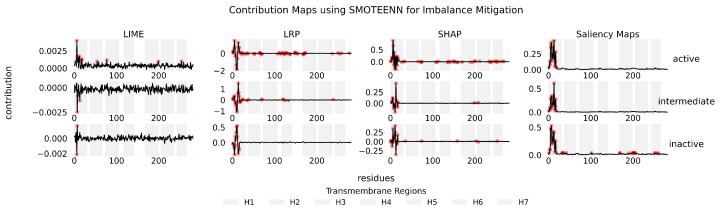
Contribution maps under SMOTEENN. Relevant residues are represented as red points.

**Figure A8:**
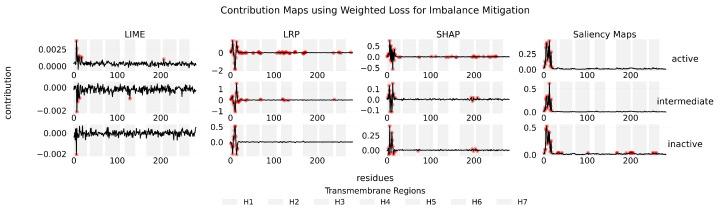
Contribution maps under Weighted Loss. Relevant residues are represented as red points.

## Data Availability

The dataset under study correspond to Molecular structures of the inactive and active states of $\beta_{2}$-adrenergic G-protein coupled receptor simulated on Google Exacycle cloud computing platform. The data are publicly available from https://simtk.org/projects/natchemgpcrdata (accessed on 1 October 2020).

## Appendix A

Here, we detail the contributions of relevant residues as identified by the LRP method, combined with the SMOTEENN technique for imbalance mitigation, which demonstrated superior performance in our experiments. Table A1 enumerates these residues for each conformational state, as determined by the Interquartile Range (IQR), and also includes their respective transmembrane regions and the computed mean contributions.

**Table A1:** Contribution of residues for different conformational states. First column: residue identifier; second column: region of the transmembrane helix; third column: computed average contribution.

**(a) Active State Contributions**		
**Residue Name**	**Transmembrane**	**Mean Contribution**
VAL31${\;}^{1.30}$	H1	0.2092
TRP32${\;}^{1.31}$	H1	0.0676
VAL33${\;}^{1.32}$	H1	0.2886
VAL34${\;}^{1.33}$	H1	0.5918
GLY35${\;}^{1.34}$	H1	1.5021
MET36${\;}^{1.35}$	H1	0.7761
GLY37${\;}^{1.36}$	H1	−0.3627
ILE38${\;}^{1.37}$	H1	−0.1454
VAL39${\;}^{1.38}$	H1	−1.3410
MET40${\;}^{1.39}$	H1	−1.7757
SER41${\;}^{1.40}$	H1	−1.8760
LEU42${\;}^{1.41}$	H1	−0.6127
ILE43${\;}^{1.42}$	H1	0.1783
VAL44${\;}^{1.43}$	H1	1.2615
LEU45${\;}^{1.44}$	H1	0.6632
ALA46${\;}^{1.45}$	H1	0.6786
ILE47${\;}^{1.46}$	H1	0.1412
VAL48${\;}^{1.47}$	H1	0.1233
ASN51${\;}^{1.50}$	H1	−0.0720
LEU53${\;}^{1.52}$	H1	0.0601
VAL54${\;}^{1.53}$	H1	0.0417
ALA57${\;}^{1.56}$	H1	−0.0615
GLU62	ICL1	−0.0552
GLN65	ICL1	0.0447
VAL67${\;}^{2.38}$	H2	−0.0353
VAL81${\;}^{2.52}$	H2	−0.0325
MET82${\;}^{2.53}$	H2	−0.0457
GLY83${\;}^{2.54}$	H2	−0.0367
ILE94${\;}^{2.65}$	H2	0.0436
LEU95${\;}^{2.66}$	H2	0.0936
MET96${\;}^{2.67}$	H2	0.0365
MET98	ECL1	−0.0449
TRP99	ECL1	−0.0800
THR100	ECL1	−0.0506
SER137	ICL2	−0.0465
TYR141	ICL2	0.0479
GLN142	ICL2	0.0565
LEU145	ICL2	−0.0627
ASN148${\;}^{4.40}$	H4	0.0412
ALA150${\;}^{4.42}$	H4	0.0840
VAL152${\;}^{4.44}$	H4	−0.0462
ILE153${\;}^{4.45}$	H4	−0.0944
MET156${\;}^{4.48}$	H4	0.0338
VAL157${\;}^{4.49}$	H4	0.0363
TRP158${\;}^{4.50}$	H4	0.0455
ILE159${\;}^{4.51}$	H4	0.0378
SER165${\;}^{4.57}$	H4	−0.0472
PHE166${\;}^{4.58}$	H4	−0.0760
LEU167${\;}^{4.59}$	H4	−0.0764
THR177	ECL2	0.0340
HIS178	ECL2	0.0583
GLN179	ECL2	0.1104
GLU180	ECL2	0.0505
ALA181	ECL2	0.0358
CYS184	ECL2	−0.0364
TYR185	ECL2	−0.0328
GLN197${\;}^{5.36}$	H5	−0.0687
ALA198${\;}^{5.37}$	H5	−0.1220
TYR199${\;}^{5.38}$	H5	−0.0586
ALA200${\;}^{5.39}$	H5	−0.0323
ILE201${\;}^{5.40}$	H5	0.0334
ALA202${\;}^{5.41}$	H5	0.0485
SER203${\;}^{5.42}$	H5	0.0526
GLN299	ECL3	−0.0397
ASN312${\;}^{7.39}$	H7	0.0489
TYR316${\;}^{7.43}$	H7	−0.0469
GLY320${\;}^{7.47}$	H7	0.0312
LEU339	C-Terminus	0.0434
**(b) Intermediate State Contributions**		
**Residue Name**	**Transmembrane**	**Mean Contribution**
VAL31${\;}^{1.30}$	H1	0.0941
TRP32${\;}^{1.31}$	H1	0.0791
VAL33${\;}^{1.32}$	H1	0.2116
VAL34${\;}^{1.33}$	H1	0.2983
GLY35${\;}^{1.34}$	H1	0.3681
MET36${\;}^{1.35}$	H1	−0.1076
GLY37${\;}^{1.36}$	H1	−0.3470
ILE38${\;}^{1.37}$	H1	−0.2240
VAL39${\;}^{1.38}$	H1	−0.5690
MET40${\;}^{1.39}$	H1	−0.8806
SER41${\;}^{1.40}$	H1	−1.0995
LEU42${\;}^{1.41}$	H1	−0.1839
ILE43${\;}^{1.42}$	H1	0.6116
VAL44${\;}^{1.43}$	H1	1.5330
LEU45${\;}^{1.44}$	H1	0.6826
ALA46${\;}^{1.45}$	H1	0.3322
ILE47${\;}^{1.46}$	H1	−0.1201
VAL48${\;}^{1.47}$	H1	−0.1765
PHE49${\;}^{1.48}$	H1	−0.1796
GLY50${\;}^{1.49}$	H1	−0.0685
ASN51${\;}^{1.50}$	H1	−0.0313
VAL54${\;}^{1.53}$	H1	0.0354
ILE55${\;}^{1.54}$	H1	0.0434
GLN65	ICL1	−0.0531
THR68${\;}^{2.39}$	H2	0.0325
TRP99	ECL1	0.0438
THR100	ECL1	0.0391
ALA150${\;}^{4.42}$	H4	0.0866
ARG151${\;}^{4.43}$	H4	0.0327
LEU155${\;}^{4.47}$	H4	−0.0390
LEU163${\;}^{4.55}$	H4	−0.0317
ASN301	ECL3	0.0491
**(c) Inactive State Contributions**		
**Residue Name**	**Transmembrane**	**Mean Contribution**
VAL31${\;}^{1.30}$	H1	−0.0437
VAL33${\;}^{1.32}$	H1	−0.0845
VAL34${\;}^{1.33}$	H1	−0.1813
GLY35${\;}^{1.34}$	H1	−0.4075
MET36${\;}^{1.35}$	H1	−0.1317
GLY37${\;}^{1.36}$	H1	0.1968
ILE38${\;}^{1.37}$	H1	0.0844
VAL39${\;}^{1.38}$	H1	0.3386
MET40${\;}^{1.39}$	H1	0.5095
SER41${\;}^{1.40}$	H1	0.5568
LEU42${\;}^{1.41}$	H1	0.1875
ILE43${\;}^{1.42}$	H1	−0.0817
VAL44${\;}^{1.43}$	H1	−0.3731
LEU45${\;}^{1.44}$	H1	−0.2599
ALA46${\;}^{1.45}$	H1	−0.2053

## Appendix B

In this appendix, we present a comprehensive collection of contribution maps computed using various explainability methods, namely LIME, LRP, SHAP, and Saliency Maps, paired with a range of class-imbalance mitigation techniques. These techniques include starting from an Unbalanced Distribution, followed by Random Undersampling, the Near-Miss algorithm, Random Oversampling, SMOTE, ADASYN, SMOTEENN, and Weighted Loss. The resulting maps are presented in Figure A1–Figure A8, respectively.

## Appendix C

Here, we list the residues consistently identified as relevant across the selected imbalance mitigation techniques, as revealed by the Venn diagrams associated with different explainability methods in Figure 9. Table A2 enumerates these residues.

**Table A2:** Contribution of common residues for different conformational states. The first column denotes the residue name, the second column indicates the region of the transmembrane helix, and the third column presents the computed average contribution.

**Saliency Maps** **Common Contributions** **Across Imbalance Methods**			
**State**	**Residue**	**Transmembrane**	**Mean** **Contribution**
active	PHE49${\;}^{1.48}$	H1	0.0337
intermediate	VAL31${\;}^{1.30}$	H1	0.0321
intermediate	VAL33${\;}^{1.32}$	H1	0.0997
intermediate	VAL34${\;}^{1.33}$	H1	0.0531
intermediate	GLY35${\;}^{1.34}$	H1	0.2772
intermediate	MET36${\;}^{1.35}$	H1	0.3054
intermediate	GLY37${\;}^{1.36}$	H1	0.3435
intermediate	ILE38${\;}^{1.37}$	H1	0.0845
intermediate	VAL39${\;}^{1.38}$	H1	0.1786
intermediate	MET40${\;}^{1.39}$	H1	0.1712
intermediate	SER41${\;}^{1.40}$	H1	0.3422
intermediate	LEU42${\;}^{1.41}$	H1	0.1551
intermediate	ILE43${\;}^{1.42}$	H1	0.6180
intermediate	VAL44${\;}^{1.43}$	H1	0.2698
intermediate	LEU45${\;}^{1.44}$	H1	0.0372
intermediate	ALA46${\;}^{1.45}$	H1	0.1233
intermediate	ILE47${\;}^{1.46}$	H1	0.2716
intermediate	VAL48${\;}^{1.47}$	H1	0.0317
inactive	GLU62	ICL1	0.0296
inactive	ARG63	ICL1	0.0361
inactive	LEU64	ICL1	0.0355
inactive	GLN65	ICL1	0.0393
inactive	ILE72${\;}^{2.43}$	H2	0.0370
inactive	TYR132${\;}^{3.50}$	H3	0.0349
inactive	ALA198${\;}^{5.37}$	H5	0.0348
inactive	TYR199${\;}^{5.38}$	H5	0.0360
inactive	SER220${\;}^{5.59}$	H5	0.0306
inactive	ARG228${\;}^{5.67}$	H5	0.0321
inactive	GLN229${\;}^{5.68}$	H5	0.0344
inactive	LEU230	ICL3	0.0336
inactive	LYS263	ICL3	0.0365
inactive	PHE264	ICL3	0.0375
inactive	CYS265	ICL3	0.0363
inactive	LEU266	ICL3	0.0338
inactive	GLU268${\;}^{6.20}$	H6	0.0322
inactive	TRP313${\;}^{7.39}$	H7	0.0343
inactive	GLY315${\;}^{7.42}$	H7	0.0317
inactive	GLY320${\;}^{7.47}$	H7	0.0296
inactive	PHE321${\;}^{7.48}$	H7	0.0323
**LIME** **Common Contributions** **Across Imbalance Methods**			
**State**	**Residue**	**Transmembrane**	**Mean** **Contribution**
active	ILE47${\;}^{1.46}$	H1	0.0014
intermediate	GLY37${\;}^{1.36}$	H1	−0.0021
intermediate	VAL39${\;}^{1.38}$	H1	−0.0012
intermediate	LEU42${\;}^{1.41}$	H1	−0.0009
intermediate	ILE43${\;}^{1.42}$	H1	−0.0009
inactive	MET36${\;}^{1.35}$	H1	−0.0021
**SHAP** **Common Contributions** **Across Imbalance Methods**			
**State**	**Residue**	**Transmembrane**	**Mean** **Contribution**
active	GLU30${\;}^{1.29}$	H1	0.0189
active	ILE47${\;}^{1.46}$	H1	0.3491
active	PHE49${\;}^{1.48}$	H1	0.0651
active	ALA57${\;}^{1.56}$	H1	−0.0165
active	PHE104${\;}^{3.23}$	H3	−0.0212
active	PHE166${\;}^{4.58}$	H4	−0.0197
active	HIS172	ECL2	−0.0158
active	GLN179	ECL2	0.0213
active	GLU188	ECL2	−0.0201
active	CYS190	ECL2	−0.0184
active	LEU230	ICL3	0.0315
active	LYS273${\;}^{6.42}$	H6	0.0180
active	GLN299	ECL3	−0.0189
active	ARG304	ECL3	0.0156
active	LEU310${\;}^{7.45}$	H7	0.0155
active	ASN312${\;}^{7.47}$	H7	0.0144
active	TRP313${\;}^{7.48}$	H7	0.0226
intermediate	VAL31${\;}^{1.30}$	H1	0.0156
intermediate	VAL33${\;}^{1.32}$	H1	0.0448
intermediate	GLY35${\;}^{1.34}$	H1	0.1313
intermediate	MET36${\;}^{1.35}$	H1	0.0838
intermediate	GLY37${\;}^{1.36}$	H1	−0.1190
intermediate	ILE38${\;}^{1.37}$	H1	−0.0173
intermediate	VAL39${\;}^{1.38}$	H1	0.0416
intermediate	SER41${\;}^{1.40}$	H1	−0.1194
intermediate	LEU42${\;}^{1.41}$	H1	−0.0501
intermediate	ILE43${\;}^{1.42}$	H1	0.1535
intermediate	VAL44${\;}^{1.43}$	H1	0.0299
intermediate	LEU45${\;}^{1.44}$	H1	0.0535
intermediate	VAL48${\;}^{1.47}$	H1	0.0164
intermediate	GLN229${\;}^{5.68}$	H5	0.0151
intermediate	LYS270${\;}^{6.38}$	H6	0.0162
inactive	VAL34${\;}^{1.33}$	H1	−0.0759
inactive	MET40${\;}^{1.39}$	H1	0.0963
inactive	ALA46${\;}^{1.45}$	H1	−0.0346
